# Spatial correlation of 2D hard-tissue histology with 3D microCT scans through 3D printed phantoms

**DOI:** 10.1038/s41598-023-45518-0

**Published:** 2023-10-28

**Authors:** Philipp Nolte, Marcel Brettmacher, Chris Johann Gröger, Tim Gellhaus, Angelika Svetlove, Arndt F. Schilling, Frauke Alves, Christoph Rußmann, Christian Dullin

**Affiliations:** 1 Faculty of Engineering and Health, University of Applied Sciences and Arts, Göttingen, Germany; 2 Institute for Diagnostic and Interventional Radiology, University Medical Center, Robert-Koch-Straße 40, 37075 Göttingen, Germany; 3 Department of Oral and Maxillofacial Surgery, University Medical Center, Göttingen, Germany; 4 Max Plank Institute for Multidisciplinary Sciences, Göttingen, Germany; 5 Department of Trauma Surgery, Orthopedics and Plastic Surgery, University Medical Center, Göttingen, Germany; 6 Department of Haematology and Medical Oncology, University Medical Center, Göttingen, Germany; 7 Brigham and Women’s Hospital, Harvard Medical School, Boston, MA USA; 8 Department for Diagnostic and Interventional Radiology, University Hospital, Heidelberg, Germany; 9 Translational Lung Research Center (TLRC), German Center for Lung Research (DZL), Heidelberg, Germany

**Keywords:** Data processing, Image processing

## Abstract

Hard-tissue histology—the analysis of thin two-dimensional (2D) sections—is hampered by the opaque nature of most biological specimens, especially bone. Therefore, the cutting process cannot be assigned to regions of interest. In addition, the applied cutting-grinding method is characterized by significant material loss. As a result, relevant structures might be missed or destroyed, and 3D features can hardly be evaluated. Here, we present a novel workflow, based on conventual microCT scans of the specimen prior to the cutting process, to be used for the analysis of 3D structural features and for directing the sectioning process to the regions of interest. 3D printed fiducial markers, embedded together with the specimen in resin, are utilized to retrospectively register the obtained 2D histological images into the 3D anatomical context. This not only allows to identify the cutting position, but also enables the co-registration of the cell and extracellular matrix morphological analysis to local 3D information obtained from the microCT data. We have successfully applied our new approach to assess hard-tissue specimens of different species. After matching the predicted microCT cut plane with the histology image, we validated a high accuracy of the registration process by computing quality measures namely Jaccard and Dice similarity coefficients achieving an average score of 0.90 ± 0.04 and 0.95 ± 0.02, respectively. Thus, we believe that the novel, easy to implement correlative imaging approach holds great potential for improving the reliability and diagnostic power of classical hard-tissue histology.

## Introduction

Pathological assessment of tissue samples is an integral part of clinical routines and preclinical research and follows well optimized and established workflows. Specimens are fixed with formalin and embedded in resin or paraffin oriented in a certain position that will determine the sectioning plane, an important consideration in both diagnostic and research histology. Consequently, the tissue is cut using either a blade or a saw for hard- or soft-tissue, respectively. Typically, microscopy analysis of these samples requires the preparation of thin sections of the specimen with a thickness in the range of few microns. Encoded through a variety of specific staining procedures targeting distinct cell subpopulations and components of the extracellular matrix (ECM), tissue-specific information is visually inspected and stored in digital images for further analysis. Despite the described specificity for cells and structures, this histological approach has the following weaknesses: (i) the specimen is destroyed in the process, (ii) the information is intrinsically 2D, (iii) due to the labor intense preparation steps, only a limited fraction of the specimen gets typically analyzed, (iv) the sectioning process cannot be effectively assigned to specific sites of interest and (v) the cutting process can introduce deformations to the tissue. Different high resolution 3D imaging methods, such as microCT, have been proposed as alternatives or additional tools to classical histology^1–3^. MicroCT allows 3D depiction of the intact uncut specimen and therefore eases the analysis of structural features. However, in conventional attenuation-based microCT the contrast is predominantly caused by differences in electron density and is therefore not specific for certain cell or tissue types. Thus, the correlation of microCT with color-coded histological images could significantly enrich digital pathology. As mentioned before, histology requires cutting of the specimen, which can introduce tissue deformation. Especially, the workflow of hard-tissue histology using a classical cutting-grinding approach yields to substantial loss of material. In order to circumvent this loss of material, the application of laser microtomy has formerly been suggested by Albers et al.^4^ for resin embedded lung specimen and was thoroughly discussed by Kunert-Keil et al.^5^ comparing classical cutting-grinding and laser microtomy for zirconia implants in rat skull bones. In short, laser microtomy, as used here, is performed by focusing a pulsed femtosecond laser in a raster scan approach into the tissue creating local micrometer sized sublimation sites, which in the end allow separating a section from the remaining bulk of the specimen. In comparison to the classical cutting-grinding technique, the loss of material is reduced. Moreover, the cutting process is contact-free and without thermal load thus allowing the generation of a stack of sequentially obtained sections. We verify our marker-based approach through multimodal co-registration and quantify the validity of the matched 2D images using a selection of similarity scores as already introduced by for instance Museyko^6^, Shafique et al.^7^ and Nagara et al.^8^.

In this study, we developed a novel workflow for spatial correlation of 2D histological slices with 3D microCT scans utilizing 3D printed phantoms as fiducial markers. We show that the implementation of these fiducial markers significantly improves precision of the sectioning process and reduces the workload in comparison to the routinely applied classical workflow of resin embedded hard-tissue histology. Furthermore, our method can be expeditiously integrated into the established routine of embedding and sectioning. The presented method is independent of histological stainings and can be employed in combination with a manifold of different samples of hard-tissue types. Correlation of the in silico microCT plane and the histological section, with a substantial high similarity score, provides additional proof that the hard-tissue histological gold standard will benefit from our extended phantom-based approach, by allowing specimen independent multimodal correlation and post-sectioning quality control.

## Material and methods

### Design and manufacturing of the cone-shaped phantoms

The cone-shaped phantoms were produced using a 3D printer (Spectrum Z510, Z Corporation), gradually affixing a calcium sulfate-based powder (zp^©^151 Powder, 3DSystems) that provided sufficient contrast against the resin embedding material in CT imaging as well as ensuring visibility independent of the staining protocol in the histological sections.

### Specimens and embedding protocol

Hard-tissue samples were obtained from different suppliers (see Table 1) and fixed in 4% formaldehyde for 2 weeks. Due to restrictions in their physical dimensions imposed by the limited field of view of the microCT (about 70 × 70 mm^2^) and the maximum sample cross-section size of 35 mm to be processed by the laser microtome, the preprocessing of the hard-tissue became a significant step in the presented workflow. First, the bone and antler specimens were cut to fit in the 25 mm wide embedding mold, dehydrated in an ascending alcohol series and finally infiltrated by a Xylene/Methyl methacrylate resin (MMA) mixture for 2 days each. Following the classic histological workflow, three phantoms were embedded in the MMA-based embedding agent (Technovit 9100, Kulzer), together with each hard-tissue specimen. In addition to the hard-tissue mentioned above, we included existing resin blocks containing deer antler and bovine femoral tissue. These existing whole blocks were embedded together with three cones in standard commercial epoxy resin, which polymerized significantly faster while being compatible with our sectioning process.

**Table 1:** List of the hard-tissue samples from different species e.g., rhesus macaques, rats, deer and cattle processed in our study. All hard-tissue specimens were obtained from animals euthanized due to veterinary reasons or from approved studies conducted either by the University of Göttingen or the German Primate Center in accordance with German animal protection laws. Bovine hard-tissue was obtained from our industrial partner LLS Rowiak, who obtained the bone from a butchery. The relevant ethical approvals are listed.

Hard-tissue obtained from	Ethical approval	Hard-tissue according to species	Embedded in	Number of slices
German Primate Center Göttingen, Germany	Bone was obtained from the German Primate Center Göttingen in accordance with the Animal Husbandry and the Pathology Unit	Rhesus macaque femur	Technovit 9100	13
Department of Trauma Surgery, Orthopedics and Plastic Surgery Göttingen, Germany	Study was approved by the district government Oldenburg, Germany (permission number 16-2358)	Rat femur	Technovit 9100	5
Department of Oral and Maxillofacial Surgery Göttingen, Germany	Study was approved by the district government Braunschweig, Germany (permission numbers: 604.42502/01-21.96 and 01-22.96)	Deer antler	Technovit 9100 and epoxy resin	10
LLS Rowiak GmbH Hannover, Germany	Tissue samples were obtained from a butchery and used for demo purposes	Bovine femur	Technovit 9100 and epoxy resin	11

### MicroCT acquisition

After hardening, the blocks were scanned in an in-vivo microCT (Perkin Elmer, QuantumFX) using the following settings: tube voltage 90 kV, tube current 200 µA, field of view 40 × 40 mm^2^ and a total acquisition time of 3 min resulted in an isotropic voxel size of 80 µm. No additional staining was required due to the strong absorption contrast of the fiducial markers. Resulting scans yielded a dimension of 512 × 512 × 512 voxels and a 16-bit gray value range. All scans were saved in the Vox1999a-Format (.vox).

### Sectioning process, staining and microscopy

In order to prepare the samples for sectioning, a priming cut was performed using a saw (Exakt 300, EXAKT Advanced Technologies GmbH). Following polishing of the cut face, the resin blocks of 25 mm in diameter were affixed to a glass slide (X-tra-Adhesive, Leica Biosystems) utilizing a specialized glue (LLS-Glue No.1, LLS Rowiak) and then sectioned using a laser microtome (Tissue Surgeon, LLS Rowiak). The resulting sections of about 30 µm thickness were stained applying methylene blue/alizarin red S staining solutions following the protocol established by Smith and Karagianes^9^. Stained sections were imaged using a microscope (Zeiss, Axiovert 200 inverted microscope) at 5 × magnification yielding a spatial resolution of 5 to 7 µm per pixel. When small artifacts such as disruptive dirt and or residual glue were present, those regions were manually removed using graphics editors such as GIMP.

### Software development

Image analysis was performed using a python 3.8.8 script based on relevant packages like NumPy 1.23.1^10^, scikit-learn 1.2.0^11^, scikit-image^12^ and OpenCV4.6.0.66^13^. Registration was performed using SimpleITK2.2.1 library^14^. Software development was realized through a python-based Jupyter 4.11.1 notebook^15^. 3D visualization was implemented using the Mayavi library 4.7.4^16^. The code is available online at https://github.com/devphilno/CT-Histology-Correlation-Phantom.

### Statistical analysis

For each completed section we computed similarity metrics based on binary classifications. Matching is only performed for the phantoms in order to circumvent difficulties in the segmentation of the tissue based on the different appearance in histology and microCT. In our case we grouped the pixels in the resulting overlay picture into three boolean subsets: true positive (TP)**—**object pixels that are present in both modalities, false negative (FN)**—**object pixels that are only present in the CT dataset, and false positive (FP)**—**objects that are pixel only present in the histological section. We considered the extracted microCT plane as the ground truth since the geometrical integrity of the specimen was not altered at this point. Based on our observations, deformations during the sectioning process did only occur to a very limited extent as analyzed by Albers et al.^4^. Pixels from the frayed edges of the cones were deducted and not factored in for the similarity score calculation. Matching quality was assessed using several different scores to opt for a better comparison to other published procedures.

Jaccard score/Intersection over Union (IoU)

1 $$IoU = \frac{TP}{{TP + FP + FN}}$$

Dice coefficient (DSC)

2 $$DSC = \frac{2TP}{{2TP + FP + FN}}$$

### Ethical approval

Hard-tissue samples from different animal species were obtained either from animals euthanized due to veterinary reasons, from studies with ethical approval in accordance with German animal protection laws or from a butchery, which is further specified in Table 1.

## Results

**Figure 1:**
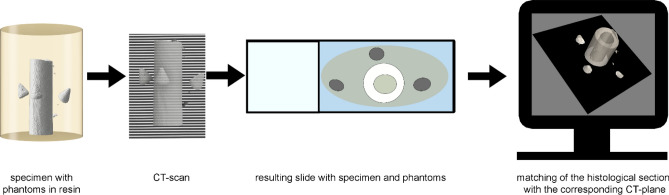
Overview of the improved histological workflow. After hardening, the resin block was scanned using a microCT resulting in a 3D digital twin volume of the sample. With the addition of three cone-shaped phantoms placed around the tissue specimen and embedded in resin the resulting image after sectioning was matched with the optimal fitting microCT plane. After sectioning, both the histological image and the microCT plane were fused using image registration algorithms.

Our novel workflow enabled the post-sectioning identification of the cutting plane for in resin embedded hard-tissue specimen and its matching into the 3D context of an a-priori performed microCT scan. Our method consisted of four fundamental steps: First, alongside with the hard-tissue, three conic phantoms were embedded in a resin block which was subsequently scanned in a microCT. Second, following the sectioning and staining process, a microscopic image of the histological slide was captured using a microscope. Third, the histological section was registered into the 3D microCT dataset using a custom-made software pipeline. This top-level description was further expanded upon in the following sections and is visualized in Fig. 1.

### General structure of the proposed workflow

**Figure 2:**
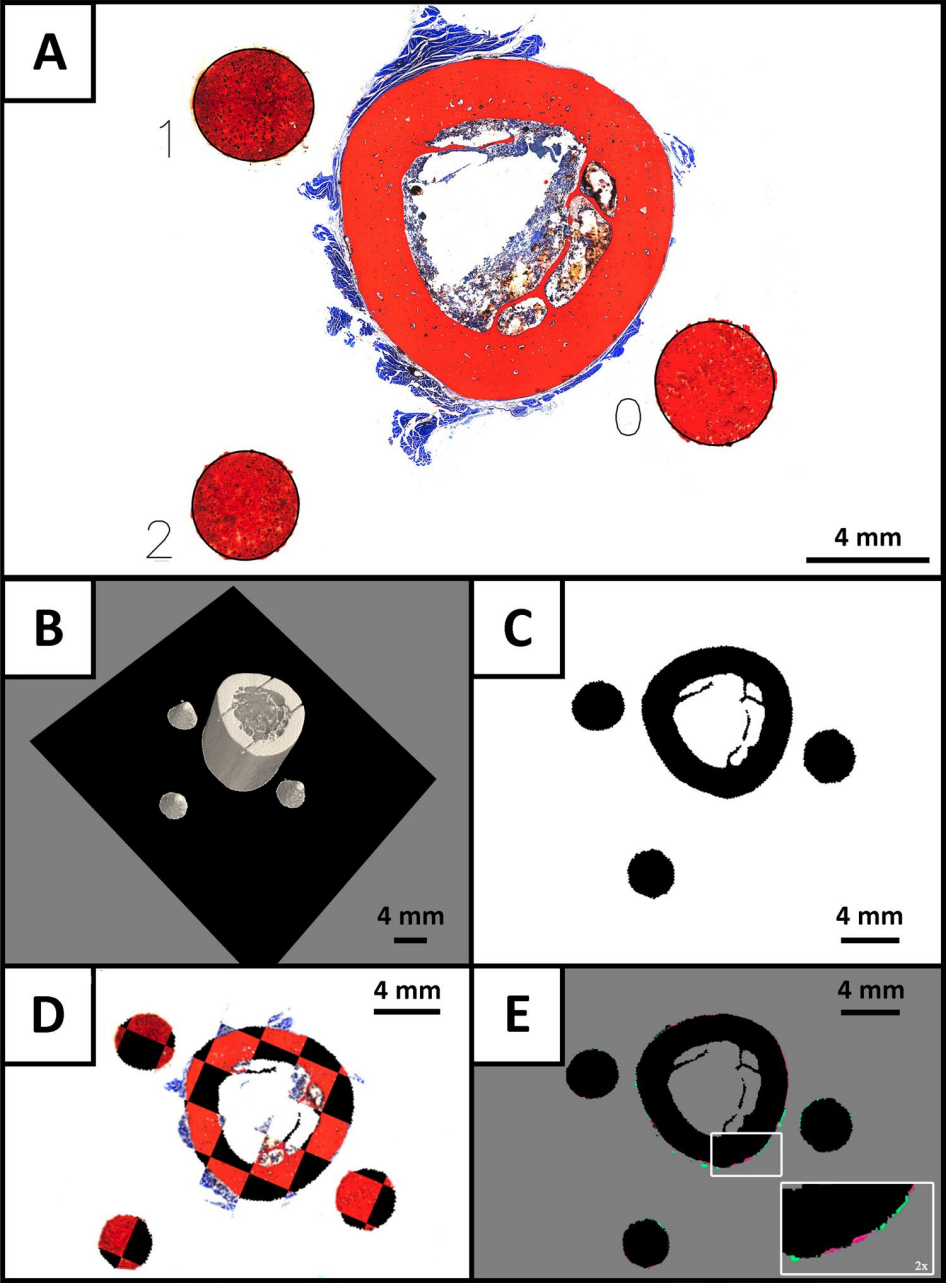
Representative images of each step of the improved histological routine for a rhesus macaque femur. After obtaining the microscopic image, the 3D printed cone-shaped fiducial markers were identified as the conic cross-section of each histological image (**A**). The lengths of the minor and major axes were calculated by fitting an ellipse in the segmented data. The length of the minor axis was used to calculate the intersection points of the cut plane with the inertia axes-system of the individual cones, resulting in three defined points (one for each cone) in the CT data coordinate system spanning the in silico cutting plane (black) in (**B**). Subsequently, the CT data in that plane was extracted and used for multimodal registration with the histological data (**C**). The qualitative result of our workflow is illustrated in the chessboard overlay in (**D**). A precise match of in silico and histological sections is evident by no visible distortions on the edges of the checkerboards. In (**E**) the sublimely matched in silico slice with the binarized histology image mask is depicted. Here, black pixels correspond to an accurate matching, while red and green pixels represent false negative and false positive samples, respectively.

All steps, such as CT scanning, physical sectioning with a saw and laser microtome, Smith–Karagianes staining of histological tissue slides in combination with imaging using a light microscope resulted in i) 2D images of the different stained histological sections as well as ii) the pre-cut 3D representation of the specimen. These datasets served as input for our cone-based workflow. We deliberately chose conic phantoms as the shape and size of the elliptic cross sections in the histological images allowed calculating the cutting plane position through these fiducial markers. In combination with the a priori knowledge of the cone dimensions this enabled us to calculate the plane intersection in the cone-coordinate system. The combined information of the estimated cut positions of the three marker objects were used to define the position and orientation of the histological section within the 3D context of the microCT dataset. The preceding consecutive steps are depicted in Fig. 2A–E and described in detail in the following sections. First, the sectioned 3D printed phantoms, now presented as circular or elliptic cross-sections respectively, were identified in the histological section (Fig. 2A). Each fiducial marker, i.e., conic section was addressed by an index ranging from 0 to 2. A detailed description of the processing pipeline for the histological section is provided in “Retrieval of cutting parameter from the histological data”. Based on the geometrical parameters of the sectioned phantoms an in silico digital twin of the physical cutting plane was computed and identified in the microCT volume (Fig. 2B as described in “Computation of the in silico cut plane parameters in the microCT dataset”). The in silico cutting plane was then extracted (Fig. 2C) and registered with the histological section (Fig. 2D). In a final step the quality of the overlay was assessed using the delineated similarity metrics IoU and Dice score (Fig. 2E). A qualitative depiction of the validity of the proposed workflow is described in “Calculation of the in silico cutting plane and registration of the data”, while a statistical evaluation of the computed similarity scores is presented in “Validation of the extracted in silico microCT plane through multimodal registration with the histological section”.

### Design of the software pipeline

**Figure 3:**
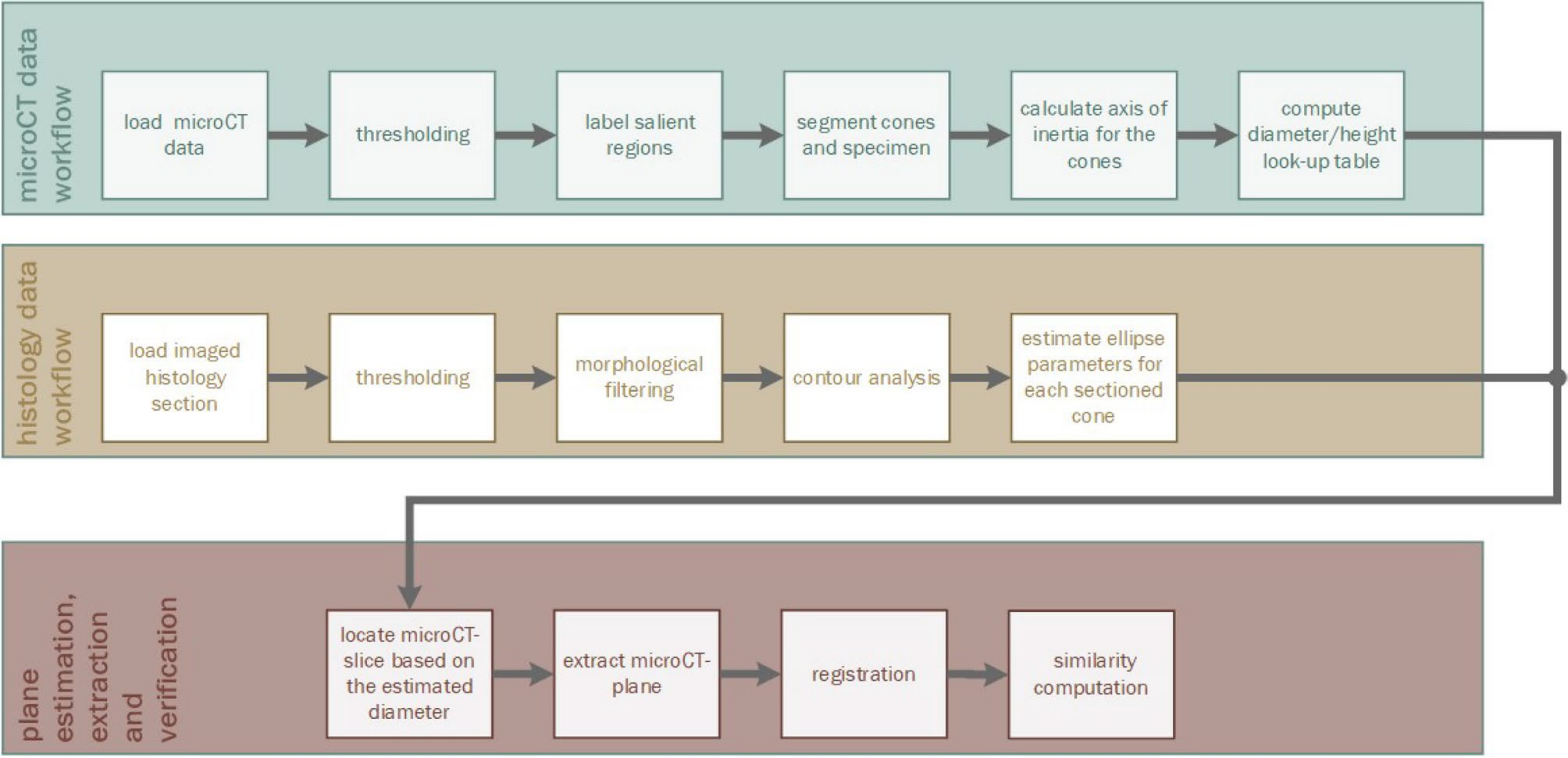
Visualization of the functional steps realized to retrieve the physical cutting plane of the sectioned hard-tissue by the created software. The process was split into two information retrieval scripts to extract the position of the marker objects from microCT-Volume (green) and the ellipse parameters from the post-sectioning histological image (gold). By correlating both modalities in the red pipeline, the extraction of the in silico cut plane and the subsequent verification of the process through 2D → 2D registration was realized. The resulting match of in silico cut plane and histological image was then quantified using similarity scores.

The developed software was structured in three main parts as depicted in Fig. 3. In both microCT (green pipeline) and histological data (gold pipeline) the 2D or 3D representations of the cones were extracted, and the obtained information was fused in a joint pipeline (red) allowing the computation of the in silico cutting plane in the microCT dataset. This plane was then registered to a binary mask extracted from the subsequently generated histological section. To evaluate the validity of the match similarity scores were calculated. Each of the displayed dedicated pipelines are thoroughly described in the following sections. An overview of the functional steps realized to retrieve the physical cutting plane of the sectioned hard-tissue is shown in Fig. 3.

#### Computation of the in silico cut plane parameters in the microCT dataset

**Figure 4:**
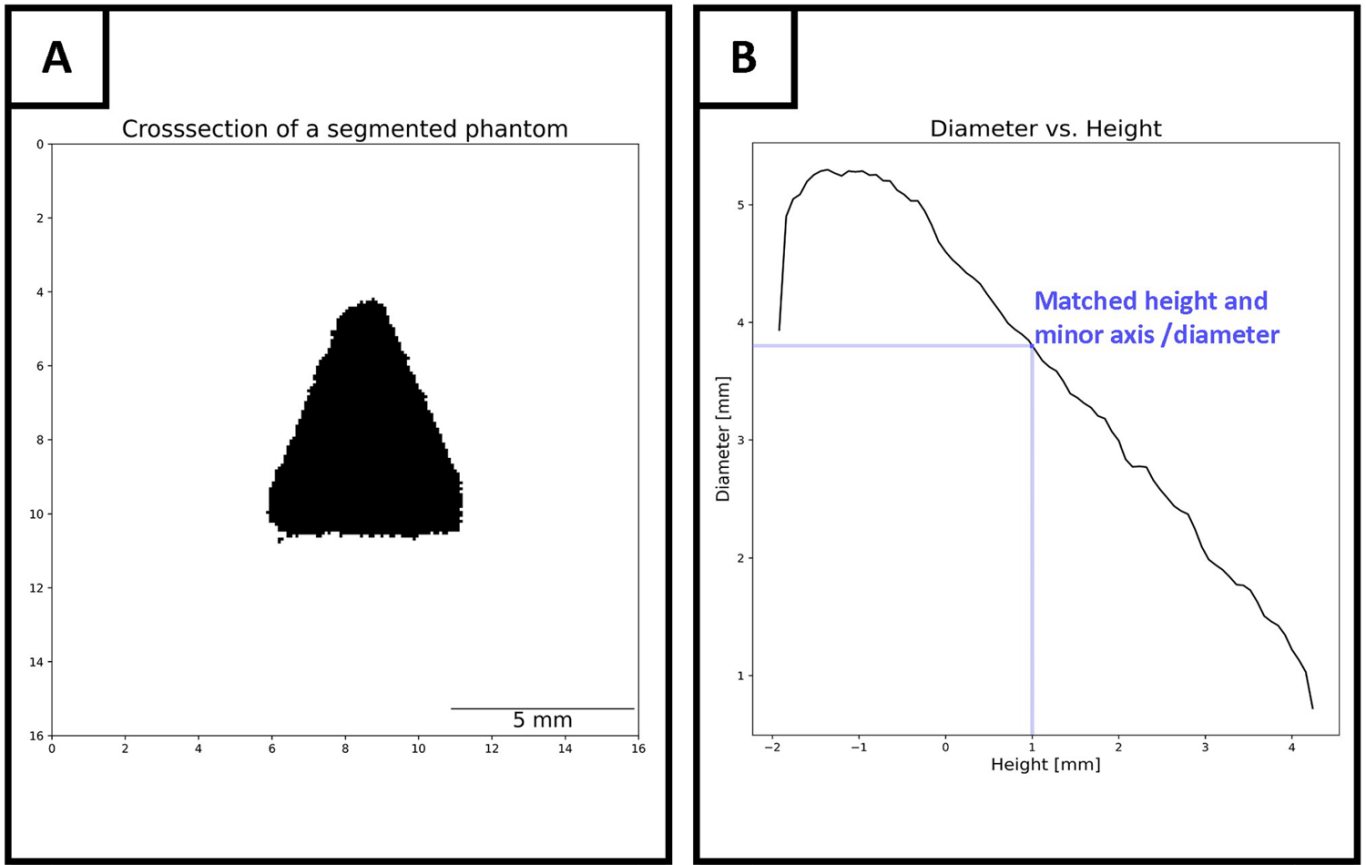
Calculation process of the relationship between phantom’s diameter and height. The segmented pixels (black) for each line were computed and stored (**A**). The index corresponding to the image-matrix column and the number of black pixels were scaled according to the CT’s resolution and consequently matched. A proportional increase of the diameter traversing from apex to base of a cone is shown in (**B**), thus each diameter can be assigned to a certain height. After smoothing, the resulting height was normalized using the known center of gravity as origin and saved in a diameter/height lookup-table.

After scanning the resin block, containing hard-tissue and phantoms, thresholding and segmentation were performed. Based on the different intensity ranges for both phantoms and specimen, we succeeded in isolating the hard-tissue and cones based on saliency. Following the thresholding, each group of adjacent voxels with similar intensity was assigned to different labels using the *SciPy* label-function. The resulting list of labels was sorted by the number of voxels they enclose. In this study the largest object was the specimen, while the next three largest objects represented the three phantoms. For each segmented cone, the center of gravity as well as the centralized covariance matrix was computed. By solving the Eigenvalue problem of the before mentioned covariance matrix the axis of inertia was obtained by selecting the Eigenvector yielding the largest Eigenvalue. Since the center of gravity was now possible to address and the axis of inertia was known, the 3D cone was sliced along the Eigenvector, allowing to obtain the center cross-section. Therefore, no specific arrangement of the cones in the resin block is required. However, the approach is limited to the fact that the desired plane must intersect all three phantoms. The resulting image, depicted in Fig. 4A, shows the frontal plane of the cone. The salient pixels were summed up iteratively for each row and the result was scaled according to the resolution of the scan, thus resulting in a look-up table depicting the relationship of the diameter per slice. This process was repeated for each cone due to random alterations occurring during the printing process, thus allowing for robust retrieval. Since the CT scan was based on isotropic slices, matching both diameter and height is trivial. A plot of the relationship between diameter and height is depicted in Fig. 4B.

The relationship between diameter and height was consecutively computed and mapped for each phantom. All voxels belonging to a given phantom were identified by the three-dimensional voxel coordinates from which the geometrical mean yielded the center of gravity. Since this point can be robustly identified in 2D and 3D, it was possible to match the computed minor axis length, obtained from the histological section, with the height/diameter lookup-table and thus enable the identification of three points on the respective axes of inertia of each cone which lies in the presumed in silico cutting plane. The height variable was centered around the center of gravity thereby facilitating a resilient approach to addressing each individual section. As this reference point was already established, a robust mapping to the 3D space was realized. The resulting graph in Fig. 4B was smoothed using a third-degree polynomial regression function and implemented through a *Savitzky-Golay filter*^17^ from the *SciPy* python package^18^. Following the completion of the CT data workflow an inference process for the histology data was implemented as described below.

#### Retrieval of cutting parameter from the histological data

Aiming to match images of both modalities, the post-sectioning RGB microscopic image of the histological slide was first split into its three-color channels. This resulted in three gray-scale variants of the histological section. The channel, which demonstrated the strongest contrast for the hard-tissue as well as for the marker objects was further utilized. This image was then binarized using *Otsu’s method*^19^ (*scikit-learn*) for thresholding. Next, a morphological filtering pipeline was employed to correct for insignificant noise and artifacts such as holes, resulting in a binary mask, suitable for the registration with the in silico microCT plane. To assure accurate detection of the elliptic cross-sections of the fiducial markers, the image was first dilated with a disk-shaped structural element with a size of 5 pixels to close holes, which might be present in the cone-shaped markers or in the hard-tissue. All significant parts of the image were now represented as closed and separate objects. This was followed by the removal of small undesired objects like noise and/or a scale notation using an erosion filter with a disk kernel of 30 pixels in diameter. In a final step, morphological erosion and dilatation filters were applied after another to correct for possible holes in the imaged hard-tissue or cones. Following the erosion, the *binaryfillholes*-function (*SciPy* library) with a square structuring element of 20 pixels per edge was applied. In a final subsequent application of a morphological erosion and dilation, with a disk-shaped structuring element with a size of 5 and 25 pixels respectively, remaining noise was removed. Undesired ablation of pixels belonging to the phantoms was accounted for simultaneously. Optimization of the defined parameters may be required in accordance with the processed image. However, the chosen parameters showed great comparability with our data and were minimally adjusted in a range of 5 to 10 pixels if holes or other undesired pixel structures remained. By utilizing the *findContours*-function implemented in the *OpenCV* library^13^ all contours in the image were detected. Contours outside the expected range of the cone diameter were suppressed, which resulted in three contours visualizing the conic sections. With the *OpenCV fitEllipseDirect*-implementation the ellipse parameters were estimated for the found contours from which the minor axes of each conic section were computed. An example of the detected ellipses is depicted in Fig. 2A.

#### Calculation of the in silico cutting plane and registration of the data

**Figure 5:**
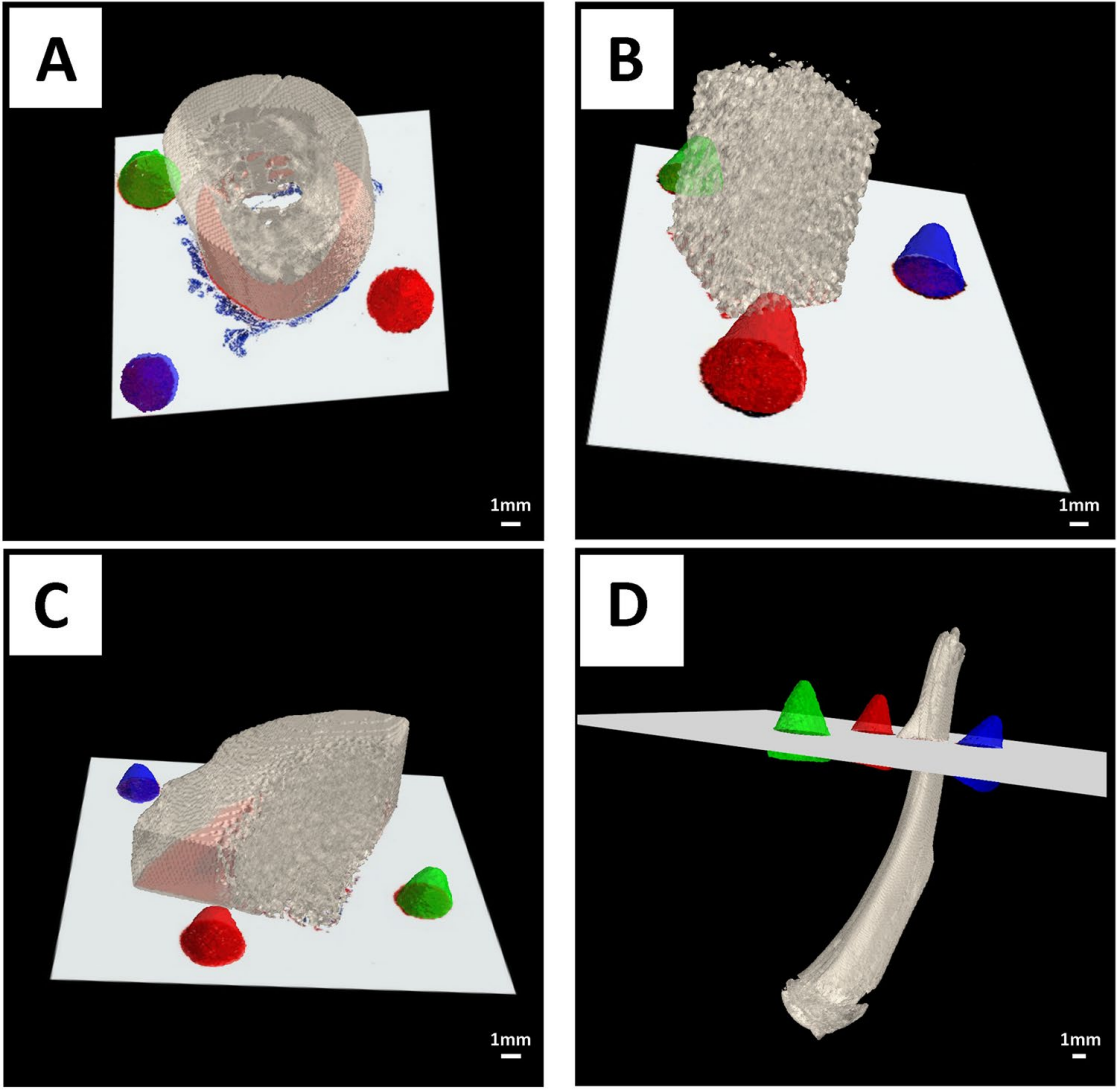
Representative images of the computed in silico 2D cutting plane inserted into the 3D CT volume performed for four hard-tissue samples from four different hard-tissue types: rhesus macaque (**A**) femurs, bovine femurs (**B**), deer antler (**C**) and rat femurs (**D**). The 3D printed cones are displayed in distinct colors (red, green, and blue), while the hard-tissue specimens were rendered using the color scheme “bone” (Hex: e3dac9 https://encycolorpedia.com/e3dac9).

With the completion of the width vs height mapping, it was now possible to identify the position at which each cone was cut. By matching the determined minor axis length with the corresponding computed diameter from the CT scan, three points were defined in the volume through which the physical cut was performed (Fig. 2B). On the basis of these points, a cutting plane was extracted (Fig. 2C). In a final step, both the histological image and the microCT plane were matched by means of rigid image registration implemented by the simpleITK library^14^ (Fig. 2D). To allow for a qualified match, we downscaled the histological image to the resolution of the microCT data. Figure 5 qualitatively illustrates the validity of the plane estimation and alignment workflow. The described workflow was conducted for hard-tissue samples of rhesus macaque femurs (Fig. 5A), bovine femurs (Fig. 5B), deer antler (Fig. 5C) and rat femurs (Fig. 5D) in order to ascertain the potential applicability of our method across a broader scope of specimens. The cone-shaped fiducial markers in Fig. 5 are displayed in different colors. The digitalized histological section was inserted into the in silico cutting plane, thus visualizing the multimodal overlay. The precise matching was demonstrated with the insertion of the 2D histological section in the 3D microCT volume. Excess soft-tissue present in Fig. 5A was clearly visible and could not be matched with the microCT data due to its lack of x-ray contrast.

### Validation of the extracted in silico microCT plane through multimodal registration with the histological section

In order to validate the accuracy of the presented workflow, both the histological section and the in silico cutting plane extracted from the microCT scan were co-registered. The accuracy of the overlay and subsequently the accuracy of the estimation of the in silico plane was computed using the defined IoU and DSC. We conducted the proposed workflow for four different types of hard-tissue specimen of different species as depicted in Fig. 5. After a priming cut using a pathological saw and further sectioning by a laser microtome 39 sections were obtained which were then stained using the Smith-Karagianes protocol for microscopical analysis. For each section, a similarity score was computed of which we calculated the mean and standard deviation. The results are presented in Table 2.

**Table 2:** Comparison of the final alignment output produced by our script. Overlays of both the computed candidate microCT plane and the binary mask were obtained through thresholding. Removal of artifacts was realized through morphological filtering. The resulting image was color-coded with black pixels yielding true positive data points, while green and red represent false positive and false negatives, respectively.

Hard-tissue type	Number of slices	Mean IoU ± SD	Mean DSC ± SD
Rhesus macaque	13	0.91 ± 0.04	0.95 ± 0.02
Rat	5	0.91 ± 0.01	0.95 ± 0.01
Deer Antler	10	0.90 ± 0.05	0.95 ± 0.02
Bovine	11	0.88 ± 0.04	0.93 ± 0.03

Overlays of both the computed candidate microCT plane and the binary mask were obtained through thresholding. Removal of artifacts was realized through morphological filtering. The resulting image was color-coded with black pixels yielding true positive data points, while green and red represent false positive and false negatives, respectively (see Fig. 2E). The sum of each subset was used to calculate the similarity scores described in the “Statistical analysis” section Intersection over Union (IoU) score and Dice Score (DSC)) for the given sample. For each hard-tissue type we computed the mean and standard deviation of the achieved score.

Comparable high scores independent from the specimen types resulted from our experiments. Only in the case of the bovine specimens slightly lower scores of 0.88 IoU and 0.93 DSC were calculated.

## Discussion

This study shows that the inclusion of 3D printed cone shaped phantoms as fiducial markers enables a fast and accurate matching of subsequently generated 2D histological sections to the corresponding in silico planes in 3D microCT datasets of resin embedded specimens. Since the proposed workflow generates the relationship between the two modalities based on the contrast of the marker objects only, it is independent of specific staining protocols. In addition, the demonstrated precise co-registration allows for the local fusion of 3D measures from microCT with the information about specific cell types found in these regions.

Fiducial markers being co-embedded in hard-tissue histology have already been used for instance by Rau et al.^20,21^, who inserted artificial markers in the form of cylindrical pins, in order to register adjacent tissue sections. Their approach needs to preserve the order of the sections and requires the use of special grinding equipment. The application was further limited by a dedicated preparation step, in which the pins are inserted into holes drilled using a computer numerically controlled (CNC) mill. Other researchers suggest the use of individual 3D-printed slicer-molds to aid and guide the cutting process^22^. Here the specimens were scanned, segmented, and loaded into a CAD Software. A mold was created with the addition of lamellar structures allowing for the manual parallel sectioning using a microtome. A similar approach was investigated for renal cancer specimens by Crispin-Ortuzar et al.^23^ which relied on the manual definition of dedicated anchor points to align the tissue specimen inside the mold. For both processes a dedicated mold had to be created for each tissue specimen, thus hampering universal application of only one mold for different specimens. The porous nature of the here used calcium sulfate-based phantoms present a major limitation. While soaking uncured prints in glue beneficially influences the durability, we can clearly observe arbitrary structures around the perimeter of the sectioned cones in 2D or the surface in 3D scans. Laser microtomy still requires minimal manual polishing of the cut resin block and section, which could cause partial or complete destruction of loosely clustered phantoms. This may hinder the correct correlation of microCT plane with a histological section. Despite the fact laser microtomy is advantageous due to minimal material loss, it limits the approach to resin embedded specimens with a cross-section of less than 50 × 50 mm^2^. In the future, one possible solution to this problem is to further investigate other 3D printable materials that yield sufficient contrast while being resistant to the abrasive process Identification of the cones and matching with their in silico counter parts, segmented from the microCT scan is up to now designed as a manual process for now. In due course we aim to automate this through color-coding of the printed cones and the addition of geometrical identifiers like unique toppers or an additional marker with a unique shape. By validating the extracted in silico microCT planes through multimodal co-registration with the corresponding histological sections, we achieved an average IoU score of 0.90 ± 0.04 and a Dice score of 0.95 ± 0.02 in four different cases. This points to a good and reproducible degree of image overlay. In object detection and segmentation applications a IoU score of 0.9 and higher, as achieved here, is considered to be very good. The results obtained in terms of DSC, might be due to the better generalization and lesser penalization of smaller mismatches in comparison to the IoU score. In the case of bovine bone samples, which contained a higher percentage of trabecular structures (as shown in Fig. 5B), our method performed moderately worse compared to the other hard-tissue types. Even though we analyzed only five samples of rat tissue we believe that our approach produces consistent and representative results across all investigated hard-tissue types. Since we rely on Boolean metrics to express the achieved similarity, we are not providing deviations in commonly used distance units, thus allowing a comparison of performance independent of imaging resolution as discussed before^24^. Rau et al.^21 reported a deviation of 0.0 to 0.4 mm when comparing grooves in generated sections and CT data, which would correspond to up to 5 pixels in our method.^ Sandgren et al.^22^ noted an median in-plane offset of 1.8 mm for the use of 3D printed slicers based on MRI scans. This could be a motivation to apply our phantom approach in an MRI context. In a similar slicer application^23^ a maximum DSC of 0.96 was achieved when comparing manually contoured photographs of the sectioned tissue with their MRI segmentation. In contrast, applications matching histology with microCT scans achieve a similar performance. Nagara et al.^8^ matched lung specimen sections with an a priori CT scan and reconstructed an in silico volume of the specimen based on non-rigid registration. They achieved a maximum DSC of 0.8 and a IoU score of 0.667. Since our cone-based workflow performs significantly better than the so far published results, we see great potential for its use in 3D virtual histology. The 3D printed cones make our workflow highly accessible and easily reproducible, while demonstrating high contrast, even when embedded in resin or similar polymers. The improved marker-based registration process is independent from additional staining since the fiducial markers intrinsically generate enough contrast in light microscopy. We show that the here used different types of hard-tissue did not affect the computed results. Since the specimen itself was not part of the registration process the presented approach is also independent of the actual shape or size of the specimen applied. The phantoms can be inserted with the specimen during embedding or be re-embedded later together with the polymerized resin block. Neglecting the 3D printer and the printing material, the described marker-based registration process does not rely on dedicated grinding equipment and does not require individual cutting guides such as 3D printed slicers and can therefore be easily integrated in existing workflows. The conic shape of our markers and the resulting elliptic cross-sections enable a direct calculation of the actual cutting plane, without the need of any additional knowledge. Nevertheless, this limits the approach to cutting angles and cutting ranges that intersect with all the three markers. Moreover, damaging the shape of the cut cone during the polishing process of the slide must be avoided. Currently, our approach is limited to using cones printed with calcium sulfate since achieving sufficient contrast in microCT imaging is essential. The accuracy of this printing technology affects the outcome of the registration and does not allow to reduce the size of the markers dramatically. We also tried conventional fused deposition modeling and stereolithography printing methods, however the resulting phantoms were either not compatible with the embedding procedure or showed no sufficient contrast in microCT imaging.

In combination with the presented similarity measures between the physical (histological) plane and the in silico (microCT) plane, the approach can also be used as a feed-back loop for automated CT guided sectioning techniques.

## Conclusion

Here we demonstrate a novel approach to simplify and extend the multimodal matching process for hard-tissue histology and microCT. Possible applications are the 3D reconstruction of an in silico histological volume through subsequent reconstruction of the resulting conic sections. While there is potential for optimization, we believe that the inclusion of our cone-based extension to the classical histological workflow into the efforts of other research projects to assess structural features in hard-tissue sections and their addition to it will enrich the information gained by conventional histopathology. Furthermore, we expect that our method could enrich efforts aiming to reconstruct a 3D virtual histology volume, by eliminating the need for corresponding blockface or en-face images or costly matching-candidate-algorithms. Through our method we see a clear benefit to ease the complex fusion of microCT and histological imaging.

## Data Availability

The datasets analyzed during the current study are available from the corresponding author upon reasonable request.
